# An Intervention Using Gamification to Increase Human Immunodeficiency Virus and Sexually Transmitted Infection Screening Among Young Men Who Have Sex With Men in California: Rationale and Design of Stick To It

**DOI:** 10.2196/resprot.8064

**Published:** 2017-07-17

**Authors:** Christopher M Mejia, Daniel Acland, Raluca Buzdugan, Reva Grimball, Lauren Natoli, Mark R McGrath, Jeffrey D Klausner, Sandra I McCoy

**Affiliations:** ^1^ Public Health Division AIDS Healthcare Foundation Los Angeles, CA United States; ^2^ Goldman School of Public Policy University of California Berkeley, CA United States; ^3^ School of Public Health University of California Berkeley, CA United States; ^4^ David Geffen School of Medicine University of California Los Angeles, CA United States; ^5^ Fielding School of Public Health University of California Los Angeles, CA United States

**Keywords:** gamification, men who have sex with men, young men who have sex with men, HIV screening, STI screening, self-determination theory, game design, game elements, incentives, intrinsic motivation, homosexuality, male, human immunodeficiency virus, sexually transmitted diseases

## Abstract

**Background:**

In the United States, young men who have sex with men (YMSM) remain disproportionately affected by human immunodeficiency virus (HIV) and other sexually transmitted infections (STIs). Although routine HIV/STI screening is pivotal to the timely diagnosis of HIV and STIs, initiation of appropriate treatment, and reduced onward disease transmission, repeat screening is underused. Novel interventions that incorporate elements of games, an approach known as gamification, have the potential to increase routinization of HIV/STI screening among YMSM.

**Objective:**

The study aims to test the hypothesis that an incentive-based intervention that incorporates elements of gamification can increase routine HIV/STI screening among YMSM in California.

**Methods:**

The study consists of a formative research phase to develop the intervention and an implementation phase where the intervention is piloted in a controlled research setting. In the formative research phase, we use an iterative development process to design the intervention, including gathering information about the feasibility, acceptability, and expected effectiveness of potential game elements (eg, points, leaderboards, rewards). These activities include staff interviews, focus group discussions with members of the target population, and team meetings to strategize and develop the intervention. The final intervention is called Stick To It and consists of 3 components: (1) online enrollment, (2) Web-based activities consisting primarily of quizzes and a countdown “timer” to facilitate screening reminders, and (3) in-person activities that occur at 2 sexual health clinics. Participants earn points through the Web-based activities that are then redeemed for chances to win various prizes during clinic visits. The pilot study is a quasi-experimental study with a minimum of 60 intervention group participants recruited at the clinics, at community-based events, and online. We will compare outcomes in the intervention group with a historical control group consisting of individuals meeting the inclusion criteria who attended study clinics in the 12 months prior to intervention implementation. Eligible participants in the pilot study (1) are 18 to 26 years old, (2) were born or identify as male, 3) report male sexual partners, and 4) have a zip code of residence within defined areas in the vicinity of 1 of the 2 implementation sites. The primary outcome is repeat HIV/STI screening within 6 months.

**Results:**

This is an ongoing research study with initial results expected in the fourth quarter of 2017.

**Conclusions:**

We will develop and pilot test a gamification intervention to encourage YMSM to be regularly screened for HIV/STIs. The results from this research will provide preliminary evidence about the potential effectiveness of using gamification to amplify health-related behavioral change interventions. Further, the research aims to determine the processes that are essential to developing and implementing future health-related gamification interventions.

**Trial Registration:**

Clinicaltrials.gov NCT02946164; https://clinicaltrials.gov/ct2/show/NCT02946164 (Archived by WebCite at http://www.webcitation.org/6ri3G4HwD)

## Introduction

New and innovative strategies for human immunodeficiency virus (HIV) infection prevention are urgently needed to increase the uptake of health services among men who have sex with men (MSM). A growing body of evidence suggests that, under the right circumstances, financial incentives can increase the demand for HIV screening, change short-term sexual behavior, enhance linkage to care after HIV diagnosis, and promote adherence to antiretroviral therapy [1-11]. Several studies have demonstrated that incorporating elements of games into incentive-based programs—an approach known as gamification—can be more effective and cost effective than simple financial incentives alone [12-14]. Ongoing and completed studies in the United States and elsewhere, including several targeting MSM, have demonstrated the feasibility and acceptability of gamification for improving engagement in HIV infection prevention and care [15-20].

We hypothesize that gamification for HIV and sexually transmitted infection (STI) prevention may work well to reengage and motivate a new generation of MSM in a cost-effective and sustainable way. A focus on young men who have sex with men (YMSM) is particularly warranted because this population remains disproportionally affected by HIV infection. Although HIV diagnoses among gay and bisexual men have stabilized in recent years, YMSM continue to experience the greatest burden of HIV compared with any other group in the United States, with young men 13 to 24 years of age accounting for 27% of new diagnoses among all gay and bisexual men [21]. Additionally, MSM are at increased risk for STIs [22]. In 2015, 59.6% of all primary and secondary syphilis diagnosis were among MSM [22]. Rectal chlamydia and gonorrhea are also common among MSM and are associated with increased HIV acquisition [23-25]. Moreover, a growing body of evidence suggests that the Internet and social media are effective ways to share sexual health information with MSM, and YMSM in particular [26-33]. To our knowledge, at least two gamification interventions for diverse YMSM in the United States are underway or have been completed, including an intervention to reduce sexual risk behaviors (healthMpowerment) [15,16] and a mobile phone-based intervention to improve antiretroviral therapy adherence (Epic Allies) [17].

To test the potential of gamification for HIV/STI prevention, our 2-year study developed and will pilot Stick To It, an HIV/STI prevention intervention for YMSM (18-26 years of age) that incorporates elements of gamification. The objective of the intervention is to increase repeat HIV/STI screening, defined as screening at least every 3 months. HIV/STI screening is critical both as the gateway to HIV/STI treatment, which can lead to reduced onward transmission, and as a critical first step to access prevention strategies such as preexposure prophylaxis [34]. This paper describes the methodology and protocol of our study.

## Methods

The study consists of 2 distinct phases. The first phase is the formative research phase, in which we used focus groups, structured interviews, and rapid prototyping of intervention elements to determine the most effective and appropriate design for the intervention. The second phase consists of the implementation of the intervention in a controlled research design. First, we present the methodology of the formative phase and the insights gained from the different elements therein, leading to the final design of the intervention. Second, we present the protocol for the implementation phase.

### Phase 1: Formative Intervention Design

**Figure 1:**
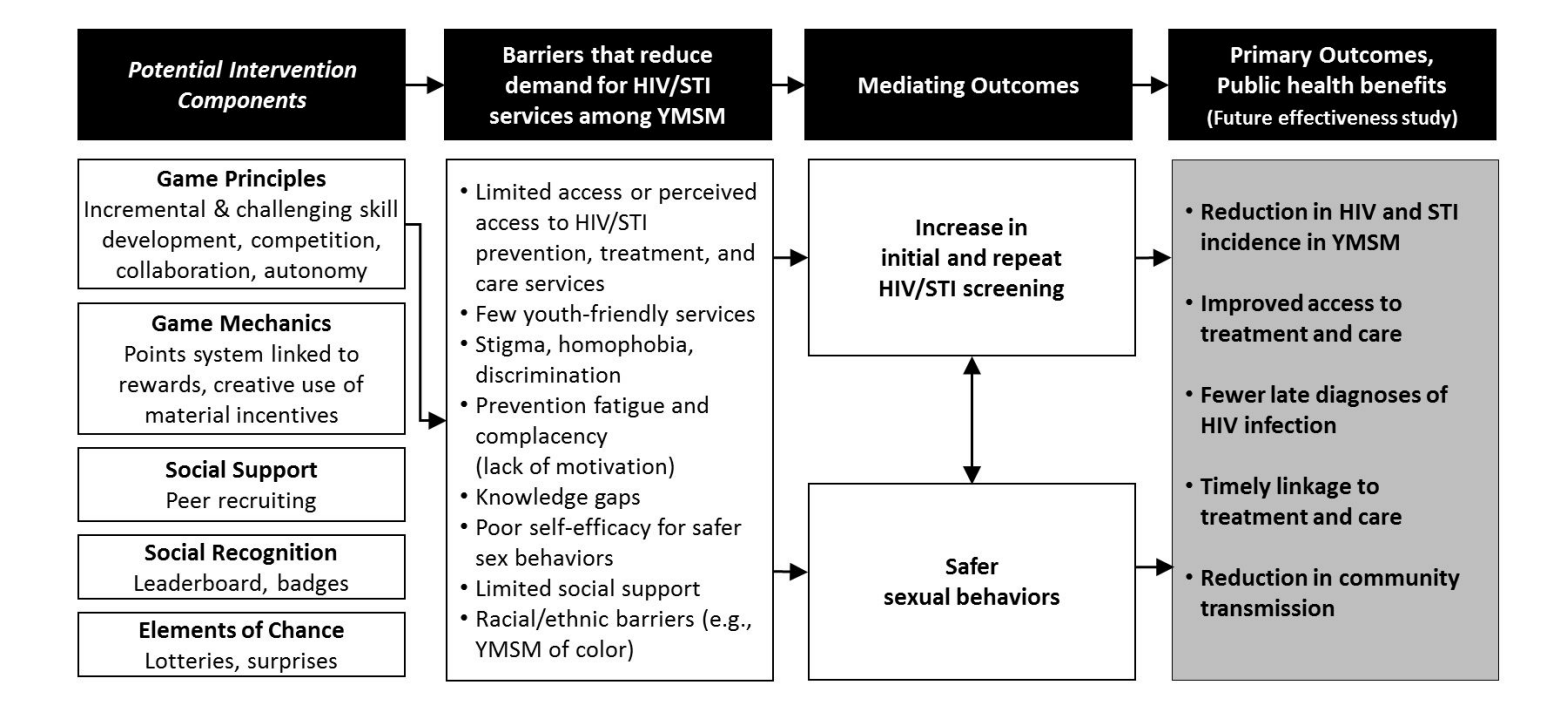
Theory of change for how a potential gamification intervention may facilitate improved health outcomes among young men who have sex with men (YMSM). HIV: human immunodeficiency virus; STI: sexually transmitted infection.

Our intervention was implemented at 2 sexual health clinics, 1 in northern California (Oakland), and 1 in southern California (Hollywood). Phase 1 formative research took place at the 2 clinics and at meeting locations on a university campus from November 2015 to July 2016. Our goal in phase 1 was to design Stick To It, an intervention incorporating gamification intended to motivate increased repeat HIV/STI screening among YMSM. Gamification, “the use of game design elements in non-game contexts,” [35] is hypothesized to amplify the motivational power of financial and nonfinancial incentives. It is informed by self-determination theory, which posits that external rewards can be internalized and generate lasting *intrinsic motivation* (defined as engaging in activities “because of the positive feelings resulting from the activities themselves”) if they are experienced in a context that satisfies three basic psychological needs: *autonomy*, *competence*, and *relatedness* [36]. Gamification scholars have argued that games and gamification can create such a context [36,37]. At the beginning of phase 1, we selected the key game elements that we intended to use in the intervention. These included a defined theme and a number of core “game mechanics,” basic mechanisms that define how the gamification intervention works. The game theme is a concept that serves to synchronize all components of the game and is critical to maximizing participant engagement [38]. Figure 1 outlines how potential gamification interventions may facilitate improved health outcomes among YMSM.

Because the formative phase of gamification design involves an iterative process of gathering information about the effectiveness of game elements and refining the intervention, we simultaneously present both the design of the formative phase of our study and the results generated by each stage of the formative research.

#### Formative Phase Methods and Results

The phase 1 research activities consisted of 4 components: (1) 10 strategy and development meetings of the entire project team, consisting of the research team, clinic staff, and a gamification consultant from the private sector; (2) 4 in-depth interviews with clinic staff about the clinic and its population; (3) 11 focus groups with 29 members of the target population (YMSM, 18-26 years of age); and (4) a series of weekly meetings of a smaller design team, consisting of 3 members of the research team and 1 of the site coordinators.

#### Strategy and Development Meetings

We held 10 meetings of the entire project team. Participants were the research team, including the project manager, the 2 site-specific project coordinators, the regional coordinators and research director of the implementation partner, and the gamification consultant. At the initial meetings, the emphasis was on broad game design-related issues such as the theme and aesthetics of the intervention design. Subsequent meetings consisted of extensive discussion of the game elements to be included, such as how to use points; whether to use a leaderboard; the frequency of participant interaction with the intervention; how much interaction between participants to include, if any; when or if to incorporate prizes; and how to use chance elements to determine prizes or other outcomes. Over time, these discussions were increasingly informed by the focus group findings. An important goal of these discussions was to integrate (1) extensive testing of game elements among the target population to determine the most suitable, engaging, and effective intervention design, as this is the cornerstone of gamification [38]; (2) knowledge of gamification and game design principles; (3) the research team’s expertise on established public health behavior change strategies, such as reminders, incentives, and education; and (4) clinic staff’s expertise on patient experience, clinic management, and overall feasibility, including the logistical realities and constrains of the participating clinics.

#### Staff Interviews

We conducted structured in-depth interviews with 4 staff members from both implementation sites. Interviewees were purposely selected to include testing counselors and clinic managers. The primary purpose of the interviews was to assess the kinds of game elements that would be feasible, appropriate, and best suited to increase engagement between clinic visits based on staff knowledge of the institutional setting and target population. The primary game elements discussed were theme and core game mechanics. The potential core game mechanics that we initially considered were (1) games of chance (eg, dice or spin wheels, gumball machines) and a system of points awarded for achievement of tasks and goals within the intervention, (2) prizes awarded based on accumulated points and outcomes of the chance elements, and (3) leaderboards that display the relative ranking of participants’ points or accomplishments and are used to create an element of competition. We used the insights gained from the staff interviews to develop the set of potential themes, core mechanics, and game elements presented at the initial focus groups. We also asked staff members to briefly pilot the games of chance in the clinic and report their perspectives about the feasibility and acceptability of each approach.

#### Focus Groups

After staff interviews, we conducted an initial set of 4 focus groups with the target population in both implementation areas. These discussions followed a standard structured format designed to elicit detailed information about preferences and characteristics, with particular focus on use of smartphones and Internet technology, as well as experiences with different types of games. Special emphasis was placed on issues of concern to race and ethnic minorities and the lesbian, gay, bisexual, transgender, questioning community. In addition, we tested the themes and game mechanics that emerged from the staff interviews with focus group participants to determine those that would be most appropriate and effective.

We conducted 7 additional focus groups, which focused more narrowly on the specific game elements of the intervention and followed a progression of increasing detail and specificity of game elements as the design and content of the intervention evolved. This stage of the formative research was intended to implement the iterative game design testing process, an important component of game testing [38]. In these focus groups, we presented mock-ups of specific game elements, in the sequence in which they would be encountered by participants in the intervention, and elicited input on each of the elements and their interaction in the overall intervention design. An additional procedure was to present multiple versions of a particular game element and elicit rankings of the alternatives in terms of potential engagement and effectiveness. While we emphasized the effectiveness of game elements and their potential for participant engagement, we also prioritized issues of sensitivity and appropriateness for the target population during focus group discussions. Namely, we aimed to ensure that the proposed game mechanics and messaging were nonstigmatizing, meaningful, and relevant to the target population. Focus group facilitators used structured focus group guides but encouraged a flexible conversation based on participant input during the sessions to facilitate friendly and open communication that allowed participants to be honest about their experiences with sexual health screenings and game design. Participant input on novel designs of specific game elements or the intervention as a whole was also encouraged.

Among the 11 focus groups held, a total of 29 participants attended, with between 2 and 9 participants per group session. The mean age of participants was 24 years old. The self-identified race/ethnicity of focus group participants was as follows: American Indian/Alaskan native: 1 (3%); Asian/Pacific Islander: 3 (10%); black/African American: 4 (14%); Hispanic/Latino: 10 (34%); white: 9 (31%); mixed or other race/ethnicity: 2 (7%).

Table 1 summarizes the key results of the focus groups: the game elements that were discussed, the main insights gained with respect to each element, and the decisions that were made about each element in the iterative design process.

**Table 1:** Focus group results: key insights and decisions about game elements to be used in the Stick To It intervention for young men who have sex with men (YMSM) in California, 2015-2016.

Game element	Key insights from focus group discussions	Design decisions for Stick To It
Theme	Prefer contemporary to retro themes. Prefer fun, lighthearted themes to highly sexualized themes. Prefer simplicity in design.	Base theme on experiences and interests of YMSM. Use bright colors, whimsical humor, and fun content.
Reminders to screen at self-scheduled time	Reminders need to be fun and rewarding. Prefer text messages (SMS^a^) over email and social media-based reminders. Frequency matters.	Send communications via SMS. Link to a fun online activity for points. Decrease communication intervals from 3 weeks to 1 week between screenings. Incorporate a “countdown timer” to remind participants when next quarterly screening is due.
Web-based activities to accompany reminders and earn points	Prefer polls and quizzes to games. Activities should not be competitive. Interest was expressed in useful information about sexual health with a fun or whimsical approach. Time is limited, so online activities should be designed to be completed in a short period of time, such as waiting in line, commuting to work or school.	Use multiple-choice quizzes, with points for answering questions, and additional points for correct answers. Limit questions to 5 per quiz. Mix informational with fun and whimsical activities. Make questions short and to the point.
Chance element for awarding prizes for screening	Guarantee participants always win at least a small prize as a means to deflect from the anxiety and stress associated with screening encounters. Prefer a combination of high-probability small prizes and low-probability large prizes. Participants need to believe the chance mechanism isn’t rigged.	Use gumball machine at the clinic as a game of chance. More points lead to more gumballs. Base prizes on color combinations of gumballs. Award small prizes for common combinations and large prizes for rare combinations.

^a^SMS: short message service.

#### Design Team Meetings

The smaller design team held a series of weekly meetings. The design team iteratively redesigned components of the intervention on the basis of the results of the other formative research components. Each meeting of the design team generated a revised set of game elements, which were then taken into the subsequent focus groups and strategy and development meetings for consideration.

#### Final Intervention Design

The final intervention consists of 3 components: online enrollment, Web-based activities, and in-person activities that occur at the clinic. Participants earn points through the Web-based activities, which they then redeem for a chance to win prizes during clinic visits. All content was developed prior to the implementation phase of the intervention.

In the first component, participants go through an online enrollment process and introduction to the intervention. The enrollment process consists of answering study eligibility questions, providing consent to participate in the study, and answering basic demographic questions. The introduction to the intervention consists of reading a short tutorial, inputting the date of last HIV/STI screening—used to set the countdown timer for the next recommended screening—and being invited to answer a 5-question multiple-choice quiz on a topic related to sexual health. Quizzes include questions that test knowledge of sexual health information that members of the target population may value (eg, STI symptoms, treatment, transmission risk, and prevention strategies) and questions primarily intended to be humorous or whimsical. Participants earn points for each step of this process (except for providing consent).

The second component, Web-based activities, occur on the intervention website (stick2it.org) and are intended to accomplish two primary goals. The first is to provide multiple reminders of the approaching quarterly screening date, which is accomplished through the prominent display of the countdown timer on the personalized webpage dashboard using a graphic presentation that was designed as part of the aesthetic theme of the intervention. The second is to provide participants the opportunity to accumulate points, which increases their chance of winning more valuable prizes at the clinic, thus increasing their motivation to receive quarterly HIV/STI screening. The online activities are primarily triggered by a series of short message service (SMS) text messages, inviting participants to visit the intervention website and complete various activities, such as taking the latest quiz, viewing their screening countdown timer, and inviting friends to join the intervention. Messages are sent with increasing frequency as the participant approaches their quarterly screening date, beginning with a 3-week interval, increasing to a 1-week interval. To complete each quiz, participants click a link in the SMS message directing them to their home page on the intervention website, where participants can also view their countdown timer. Points are awarded for answering the quiz questions, with additional points awarded for answering correctly. Participants are provided with an opportunity to take a new quiz every 3 weeks throughout the course of the intervention. In addition, participants earn points when they invite eligible friends who subsequently enroll in the intervention. Invited friends must meet all participant inclusion criteria.

**Figure 2:**
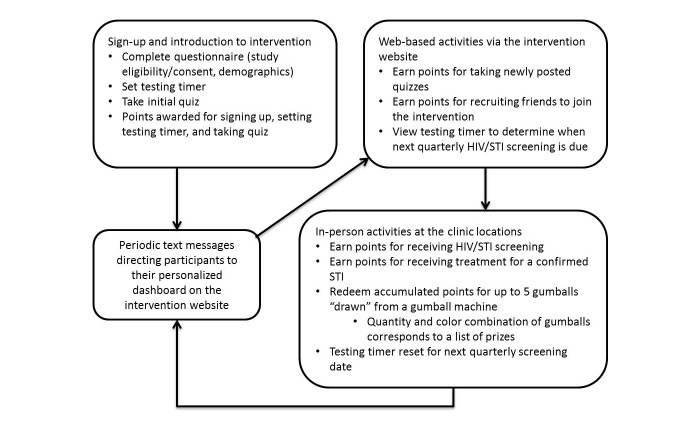
Schematic of final intervention design. HIV: human immunodeficiency virus; STI: sexually transmitted infection.

The third component of the intervention involves the game of chance and takes place at the clinic. Participants also receive additional points for visiting the clinic for screening. Participants’ accumulated points are redeemable for prizes only at the clinic (for any reason, including screening or treatment). The redemption process works as follows. Gumball machines are located at each clinic. This was both the game of chance overwhelmingly preferred by the target population through our focus group testing and the most feasible to implement at the clinics. During their clinic visit, participants can draw between 1 and 5 gumballs, depending on their accumulated points. The number of points earned corresponds with the number of gumball draws available to the participant. The color combinations of the gumballs that are drawn determine the prize received. Points redeemed for gumball draws are deducted from the participant’s accumulated points at the time of redemption. The system of points and prizes was designed to result in an average prize cost of US $5 per screening visit. After screening, the countdown timer is reset for the next quarterly screening date. Multimedia Appendix 1 presents the points system and prizes used in the intervention. Multimedia Appendix 2 presents screenshots of the intervention website. Figure 2 shows a schematic of the final intervention design.

### Phase 2 Intervention Pilot

Phase 2 will take place from October 2016 to June 2017 and will address the following aims: (1) evaluate the preliminary effectiveness of the Stick To It intervention on repeat HIV/STI screening in a pilot study, (2) evaluate the acceptability of and participant engagement with the Stick To It intervention, (3) evaluate the feasibility and cost of implementing an effectiveness evaluation on a larger scale, and (4) evaluate the cultural competence of the intervention as designed and implemented.

### Study Population and Eligibility Criteria

Our study targets individuals who (1) are 18 to 26 years old, (2) were born or identify as male, (3) report male sexual partners at the time of enrollment, and (4) have a zip code of residence within defined areas in the vicinity of 1 of the 2 implementation sites. We selected YMSM aged 18 to 26 years as inclusion criteria for this pilot study given the substantial variations in the intervention design that would be required to include adolescent-aged YMSM. Additionally, a minimum inclusion age of 18 years will ensure that participants can provide independent consent to participate in the intervention.

### Recruitment, Screening, and Sample Size

Participants will be recruited through three main channels. Multimedia Appendix 3 (flyers) and Multimedia Appendix 4 (video) present the recruitment tools used for the intervention.

#### In-Clinic Recruitment

Clinic staff will identify eligible individuals who visit the clinics for screenings, treatment, or other purposes and will inform them of the study and invite them to participate. In addition, placing flyers in the clinics and displaying the gumball machines in examination rooms will facilitate conversations about the intervention with potential participants. Those who are interested will be given a handout directing them to the intervention website, where they will be invited to complete an eligibility questionnaire and, if eligible, provide consent to participate in the study. Those with a smartphone will be invited to sign up while still at the clinic. Those who are eligible and provide consent will enter the study.

#### Community-Based Recruitment

We will place flyers in bars, other clinics, on mobile testing vans, and at selected community events. The advertisements and flyers include links to the intervention website, where individuals are invited to take the eligibility questionnaire and provide consent.

#### Online Recruitment

We will post advertisements on social networking platforms such as Facebook, Twitter, Instagram, Craigslist, and Grindr (a geolocation-based social networking app used by YMSM).

### Study Design

Owing to potential spillover effects between the treatment and control groups within local peer groups, we deemed a randomized design to be impracticable. Instead, our study will use a quasi-experimental study design with a historical control group consisting of eligible participants identified within the medical records of the same clinics over the 12 months prior to the intervention phase of the study. We preregistered the study (clinicaltrials.gov NCT02946164). All eligible individuals recruited at the clinics, at community-based events, and online will be assigned to the treatment group. We will recruit a minimum of 60 participants and a maximum of 200 participants into the treatment group and identify an identical number of individuals to serve as a historical control group. To our knowledge, this will be the first intervention using gamification to target YMSM for repeat HIV/STI screening. Accordingly, we chose a large sample size range as the upper bound, as it is not possible for us to predict what the demand will be for such an intervention among the target population.

Given the pilot nature of the study, the proposed analyses will likely be underpowered for formal testing purposes. Nevertheless, if we assume that half of the men in the comparison group will attend 1 or more repeat HIV/STI screening visits over 6 months of follow-up, with 60 men in each group, we will have 80% power to detect a minimum change of at least 27 percentage points (from 50% to 77%), which is comparable to the increase observed in an incentive-based intervention previously implemented by the community-based organization that operates the 2 clinics in our study [39].

### Outcomes

For the primary outcome of repeat HIV/STI screening, we will use routinely collected client data from the clinics’ electronic patient tracking and medical record system. A set of quantitative and qualitative secondary outcomes will be used to determine the acceptability and feasibility of Stick To It, and whether the intervention is suitable for a future effectiveness study on a larger scale. These implementation outcomes are as follows. First is the ability to recruit the study population: the percentage of eligible men approached who are willing to enroll and whether we can fully enroll the cohort within 6 weeks of study initiation. Second is participant engagement with the intervention: among Stick To It participants, we will determine (1) the proportion who successfully invited 1 or more other eligible individuals to the intervention, (2) the number of completed quizzes, and (3) the average number of quizzes participants completed. Third is cost: we will track the incremental cost of the program, relative to standard of care, which we will identify using the ingredients approach, in accordance with guidance from the World Health Organization [40]. Fourth is the potential for adverse events; that is, whether any adverse events occurred and the potential for adverse events in a larger effectiveness study. Fifth is the cultural competence of staff who implemented Stick To It services and activities and the cultural relevance of the intervention for members of the target population.

### Data Collection

For each participant, we will collect data for a period of 6 months following their entry into the study. The primary outcome is repeat HIV/STI screening, defined as the number of screenings participants receive over the 6-month observation period, an indicator of retention in the program and its effectiveness.

In addition, we will collect qualitative information about the experiences of participants and clinic staff, described below. We will use the following methods to collect our secondary outcomes.

#### Postpilot Questionnaire, Administered to All Participants

A survey offered to all participants at the end of the study period will assess HIV/STI screening at other clinics (self-reported), barriers and facilitators to screening, and perceptions of the Stick To It intervention.

#### In-Depth Interviews

We will conduct up to 10 in-depth interviews with members of the clinic staff at the end the study period. We will gather detailed information on their perceptions of the intervention, including implementation challenges and successes, adaptability and integration with clinic operations, perceptions of effectiveness, and suggestions for future improvements.

We will also conduct in-depth exit interviews with up to 40 Stick To It participants from both locations. Our goal is to gather detailed information about whether the intervention was relevant, motivating, and culturally competent. Further, we hope to assess the level of satisfaction with the process of earning and redeeming points, the rewards used, and the program’s potential applicability to others. We will purposefully select a diverse group of men from different racial and ethnic groups and men who had different levels of engagement with the intervention. Topical areas will include the participant’s perception of HIV/STI risk, perceptions about and experience with HIV/STI screening, willingness to discuss sexual health with others, particularly motivating elements of the game, and any unexpected challenges or adverse events.

We will examine whether Stick To It was culturally appropriate, relevant, and nonstigmatizing for YMSM, with an emphasis on African American and Latino YMSM, who are disproportionately affected by HIV [41]. Thus, at the completion of the in-depth interviews, we will administer a short interviewer-administered survey that includes the validated 20-item Public Perception of Physician’s Cultural Competence Scale, which measures client perceptions of physicians’ cultural competence [42]. We will adapt the scale to also refer to the clinic staff involved in Stick To It, and the design of the intervention itself. In addition, given the issues of stigma and discrimination that may adversely affect health care access among YMSM, we will include questions adapted from the validated Experiences of Discrimination [43] scale and the Discrimination in Medical Settings Scale [44] to determine whether the intervention or its implementation was stigmatizing.

In-depth interviews will be conducted in English by trained staff and will follow standard procedures [45,46]. A semistructured interview guide will cover predetermined issues (to ensure systematic data collection), but the interviewer will be free to change the sequence and wording of questions to ensure that unexpected themes can emerge [47]. Interviews will be audio recorded, with participant’s consent, and later transcribed verbatim.

**Figure 3:**
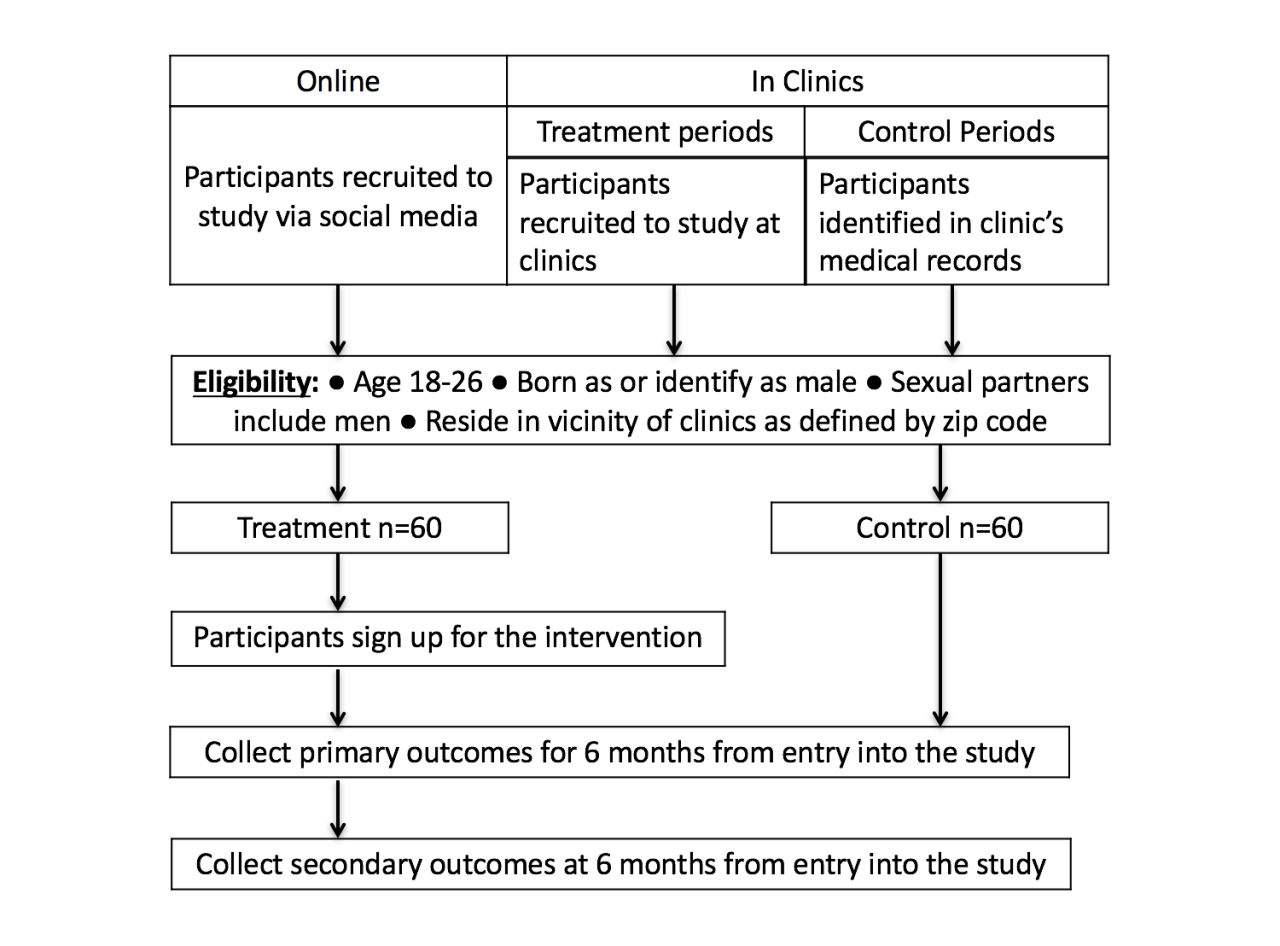
Summary of study design, screening and recruitment process, and data collection.

Figure 3 summarized our study design, screening and recruitment process, and data collection.

### Data Analysis

For quantitative outcomes, we will first assess missing data, describe participant characteristics using frequency tables, and provide descriptive statistics (eg, means, standard deviations, medians, ranges, proportions) for the primary and secondary outcomes stratified by group. For the binary outcomes, we will compare the intervention and comparison groups by computing unadjusted and adjusted prevalence ratios and 95% confidence intervals using generalized linear models, such as log-binomial regression [48-50]. For the continuous and discrete outcomes, we will use ordinary least squares and zero-inflated Poisson regression (depending on the distribution of the outcomes) to determine the change in outcomes associated with participation in the intervention, before and after controlling for baseline characteristics. All regression analyses will include robust standard errors to adjust for heteroscedasticity and clustering. We will also perform a per-protocol analysis limited to participants that had a minimum level of engagement with the intervention during the study period (eg, ≥25th percentile of points earned during the game).

Qualitative data analysis will be conducted with ATLAS.ti (Scientific Software Development GmbH) following standard procedures [47,48]. In brief, we will combine inductive and deductive techniques to strengthen the validity of the coding system and our conclusions during the multistage analysis process [46-50]. Two researchers will independently review the transcripts and code according to broad, a priori, and emergent themes related to gamification theory and barriers and facilitators of regular screening. Interviewer notes capturing contextual details, quality, inconsistencies, and impressions will also be incorporated. Data reduction will be based on coding sorts of the most central themes (eg, engagement with the intervention) followed by a systematic analysis of related themes (eg, motivation, risk perception, stigma) using coding matrices to identify relationships [44,51]. To reduce threats to validity, we will solicit feedback on our hypotheses from participants (respondent validation) [52] and rigorously examine theoretical validity—whether the data are consistent or inconsistent with the underlying theoretical models [47].

## Results

This is an ongoing research study with initial results expected in the fourth quarter of 2017.

## Discussion

The ongoing study is intended to explore the effectiveness of incorporating gamification into an incentive-based intervention to increase repeat HIV/STI screening among YMSM. Given that this population remains disproportionally affected by HIV and STIs, novel approaches that supplement proven engagement strategies may serve an important public health function. Namely, early HIV diagnosis, as achieved through repeat HIV screening, has been shown to facilitate timely linkage to medical care, initiation of antiretroviral therapy, and attainment of viral suppression, resulting in reduced transmission risk [53-55].

Given the success of nongamification interventions involving incentives, and considering the potential of gamification to harness and amplify intrinsic motivation for both short-term and long-term behavior change, we hypothesize that this approach may be particularly effective in increasing routine HIV/STI screening among YMSM. We emphasize that a fundamental principle of gamification is to enhance the motivational effectiveness of known behavior change mechanisms, such as reminders and incentives, and to amplify the existing motivation of individuals to accomplish desired behaviors. However, we do not expect stand-alone gamification interventions to replace or compete with existing types of interventions, but rather to enhance established strategies. Accordingly, we coupled gamification with an incentive-based structure for this very reason.

A secondary but important goal of this study is to explore the process of designing gamification interventions for health-related behavior change and to learn best practices for future intervention design. This is crucial, due to the fact that gamification interventions potentially require an extensive formative design process to determine the optimal set of game elements and intervention design required to incorporate such elements. One conjecture is that the success of any given health-related gamification intervention, and thus of health-related gamification more generally, may depend on highly context-specific and population-specific formative research. Thus, determining best practices for the formative phase is necessary to achieve the goal of being able to consistently and predictably design cost-effective interventions in a range of different circumstances. This paper, in addition to presenting the protocol of the study, is an attempt at documenting what formative research to develop a gamification-based intervention entails.

It is also important to recognize the limitations of this study. As previously discussed, we will use a historical control group for comparison. Although a randomized design has advantages, it was not practical because participants randomly assigned to the control group would have been exposed to some components of the intervention, such as recruitment materials posted in the clinic waiting and examination rooms (eg, comparison group contamination). Additionally, given the pilot state of the intervention, a cluster randomized design was neither warranted nor feasible. Although a historical comparison group will help us understand whether there is a preliminary signal of the intervention’s effectiveness and whether further study is warranted, it has important limitations. Most importantly, we will be unable to make causal statements about the effect of the intervention because we will be unable to adequately control for time trends between the intervention and comparison groups and the selection bias that may result from differences between the 2 groups. Nevertheless, a historical comparison group will still provide us with baseline data on repeat testing among YMSM in the same geographic region and therefore a benchmark to which we can make preliminary comparisons about the intervention’s potential effectiveness.

## Appendix

Multimedia Appendix 1 Stick To It points system/prize decoder.

Multimedia Appendix 2 Intervention website screenshots.

Multimedia Appendix 3 Recruitment tools (flyers).

Multimedia Appendix 4 Recruitment tools (video).

## Abbreviations

HIV: human immunodeficiency virus
MSM: men who have sex with men
SMS: short message service
STI: sexually transmitted infection
YMSM: young men who have sex with men
