# Supplementary material for: An Intervention Using Gamification to Increase Human Immunodeficiency Virus and Sexually Transmitted Infection Screening Among Young Men Who Have Sex With Men in California: Rationale and Design of Stick To It
Source: JMIR Res Protoc. 2017 Jul 17;6(7):e140. doi: 10.2196/resprot.8064 (PMC5537559; doi:10.2196/resprot.8064)
Supplement: Multimedia Appendix 1 [file resprot_v6i7e140_app1.pdf]

GUMBALL PRIZE DECODER

- You can redeem up to 5 gumballs per clinic visit -

1 Gumball or No Matching Colors*

*Example: or
Pick ONE color in your hand.

"Closet Cash"
AHF Swag
Gay Pride Stickers
Headphones
$5 Amazon Gift Card
$5 Starbucks Gift Card
Bottle of Lube
Glow-in-the-Dark Condom Pack

2 or more gumballs

Pair (any color)	$10 iTunes Gift Card or $10 Starbucks Gift Card
Three of a kind (any color)	$50 Cinemark Movie Gift Card
Two Pairs (any colors)	$50 Gift Card (Visa OR Amazon)
Four of a Kind (any color)	iPad Mini 2 or $250 Amazon Gift Card
Full House (any colors)	
Five of a Kind (any color)	
