# Supplementary material for: An Intervention Using Gamification to Increase Human Immunodeficiency Virus and Sexually Transmitted Infection Screening Among Young Men Who Have Sex With Men in California: Rationale and Design of Stick To It
Source: JMIR Res Protoc. 2017 Jul 17;6(7):e140. doi: 10.2196/resprot.8064 (PMC5537559; doi:10.2196/resprot.8064)
Supplement: Multimedia Appendix 2 [file resprot_v6i7e140_app2.pdf]

- An STD/HIV testing schedule you can stick to.
- Quizzes you can take a bite out of.
- Fun prizes - Now that's sweet!

JOIN SIGN IN

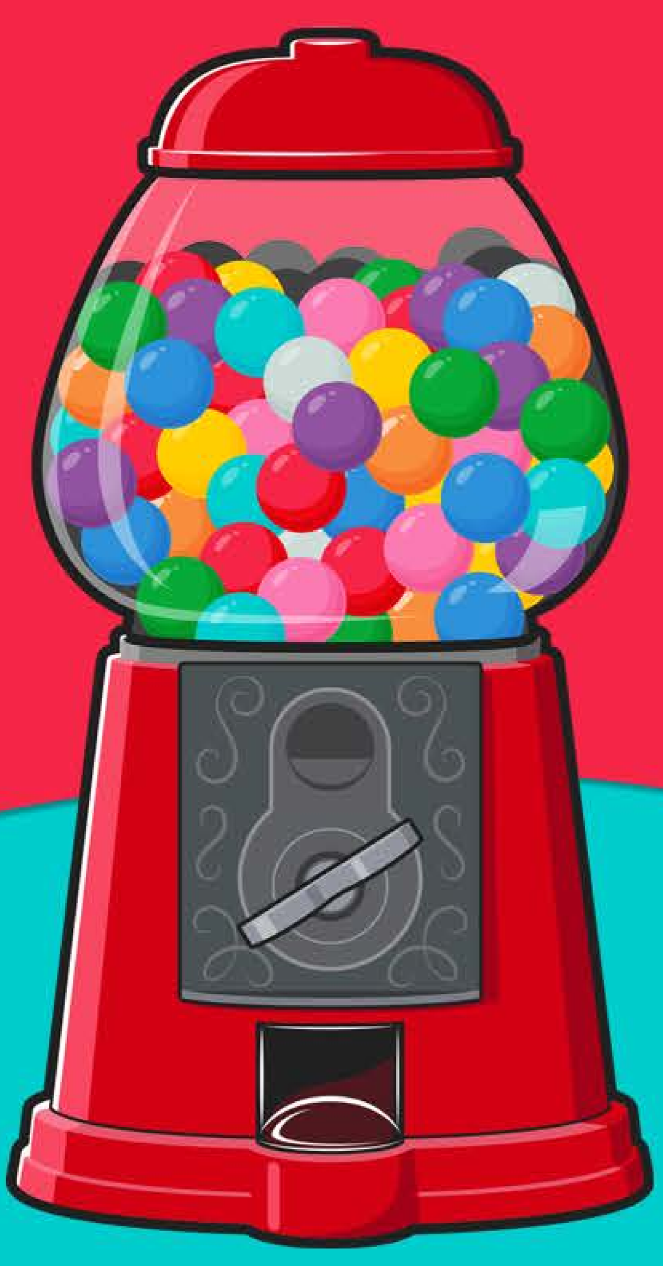

HOW IT WORKS...

SIGN UP!

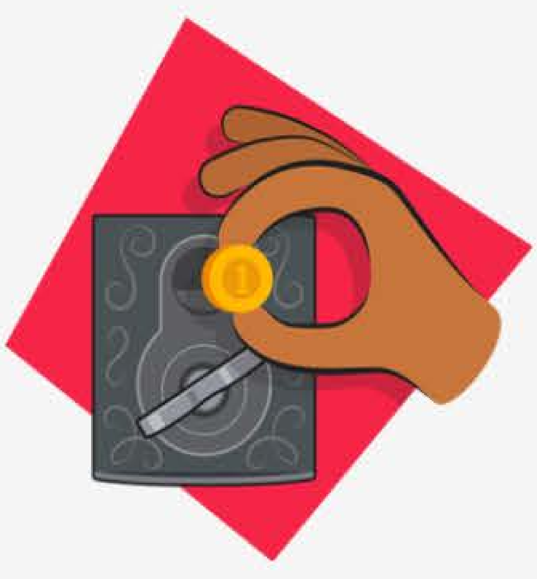

Sign up online.

Set a date for your next STD/HIV test.

We'll help you STICK TO IT.

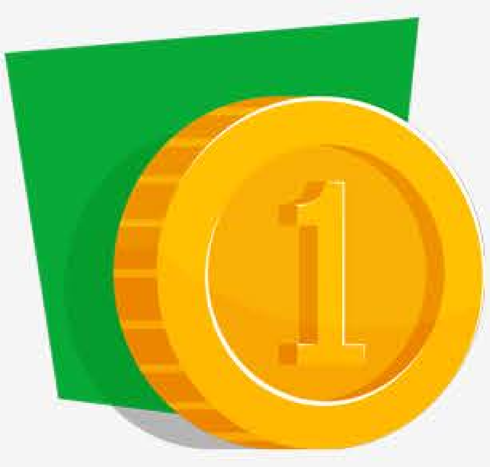

EARN POINTS!

Between now and your next test, you can earn points for responding to quizzes and for inviting friends.

GET TESTED!

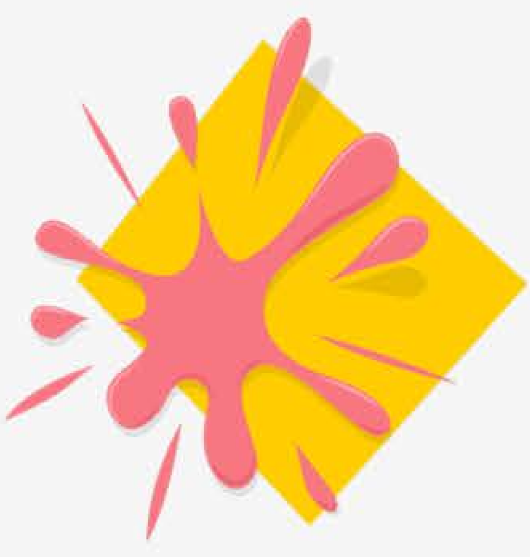

Pop into the clinic and get tested, and earn even MORE points!

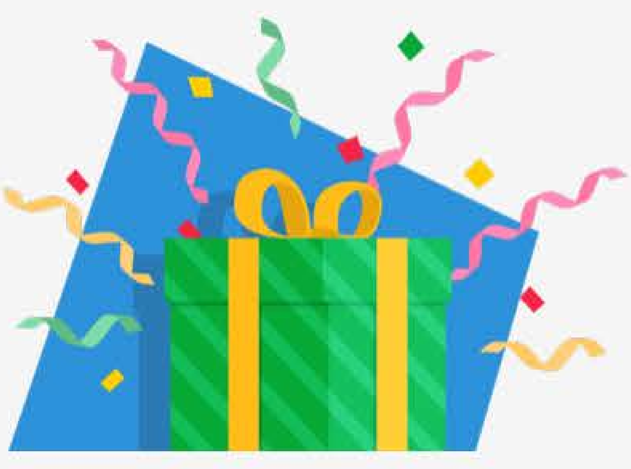

WIN PRIZES!

On test day, you can turn your points into prize gumballs at the clinic. More prize balls gives you more chances to win BIGGER prizes!

You can win prizes worth up to $250.

MORE TESTS, MORE POINTS, MORE PRIZES!

From one test to the next, we'll add new prizes and more ways to earn points.

Keep earning points, keep getting tested, keep winning prizes!

QUIZZES YOU CAN CHEW ON!

Answer quizzes to earn points between clinic visits, which gives you more choices to win.

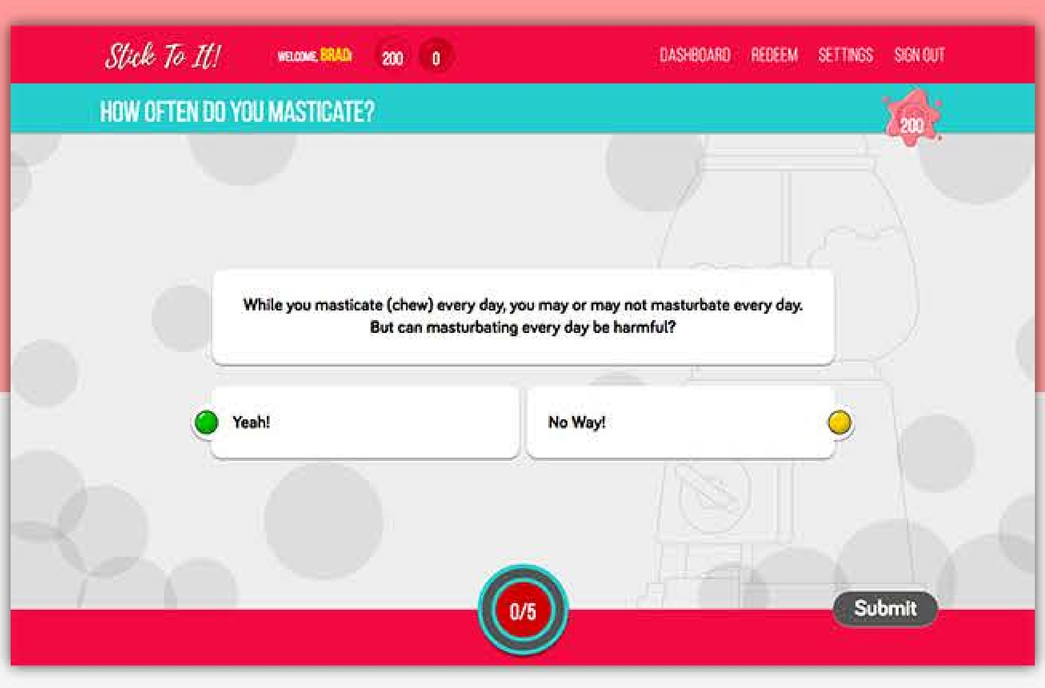

ABOUT STICK TO IT

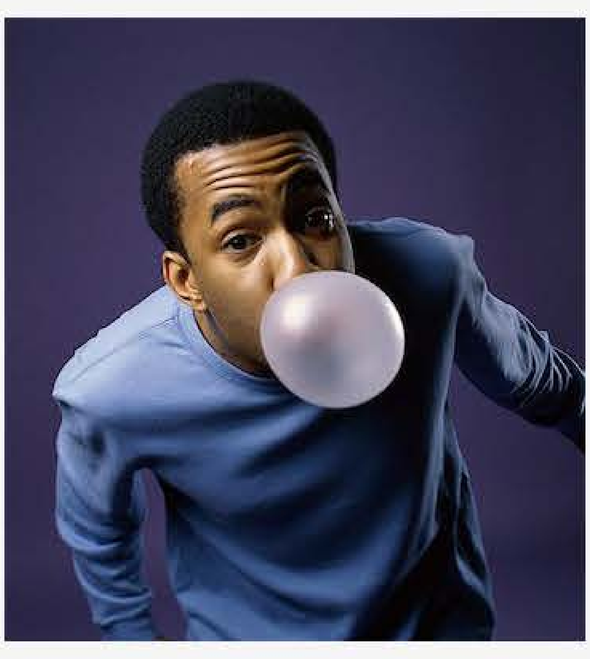

Are you having sex?

Then getting tested regularly for HIV and STDs is a fact of life. However, not only is it difficult to find time in a busy schedule, but sometimes it's hard to remember when you had your last test.

Wouldn't a program that helped you remember when to test and eased the stress of the process be helpful?

"Stick To It" is a program designed to do just that; it helps you remember when to get tested and provides a little bit of fun in the process!

We're a team from AIDS Healthcare Foundation, the University of California, Berkeley, the University of California, Los Angeles, and the Stick To It Community Advisory Board.

We're passionate about creating fun and engaging health experiences using technology and games.

While we each bring something different to the table, we have the shared goal of strengthening sexual health programs for gay, bi, and queer men in California and beyond.

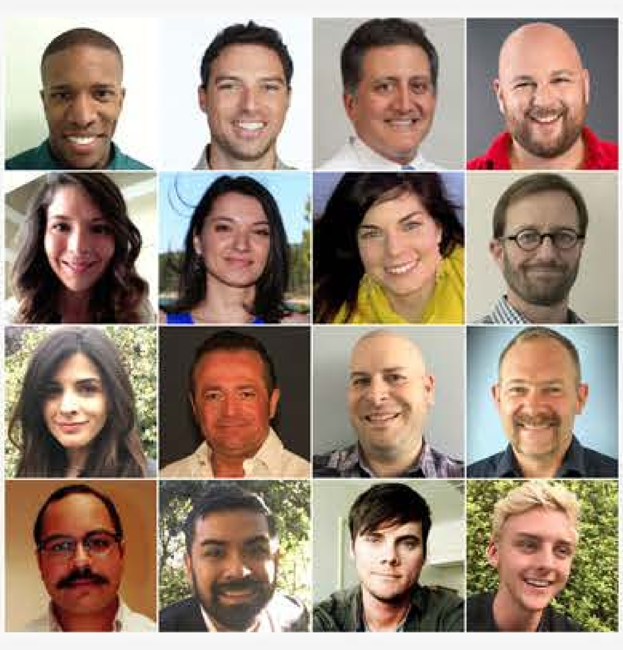

CONTACT US

If you want to know more about our team, check out our page here!

Principal Investigator:
Sandra I. McCoy, PhD, MPH
(510) 642-0513
smccoy@berkeley.edu

Project Coordinator
(Northern California):
Reva Grimball
(650) 466-8711
rgrimball@berkeley.edu

Project Coordinator
(Southern California):
Lauren Natoli
(323) 515-9586
lauren.natoli@aidshealth.org

UC Berkeley's Committee for Protection of Human Subjects
(510) 642-7461
subjects@berkeley.edu

TIME TO GET TESTED FOR HIV/STDS IN...

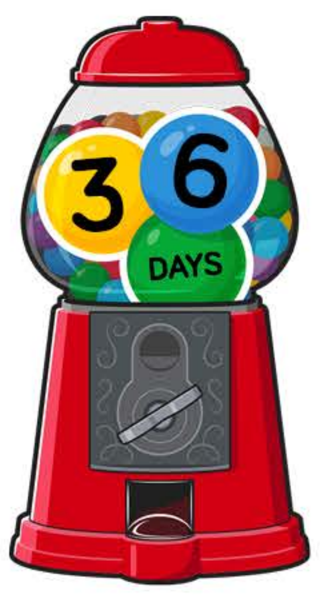

NEWS

4/24/2017

Hey, looks like you've popped into your dashboard – welcome back!

Is your timer at **zero** yet? If so, pop into the Wellness Center soon for testing! :)

This month, get **one bonus gumball** when you redeem during the week of May 8-13th, that's an EVEN BETTER chance at winning a $250 Amazon gift card or an iPad Mini 2!

Did you know our most frequent prize won is the matching pair of gumballs? That used to mean a $10 gift card, but now it means a whopping $50 movie gift card. You can treat a friend or a date to delicious snacks and the latest blockbuster!

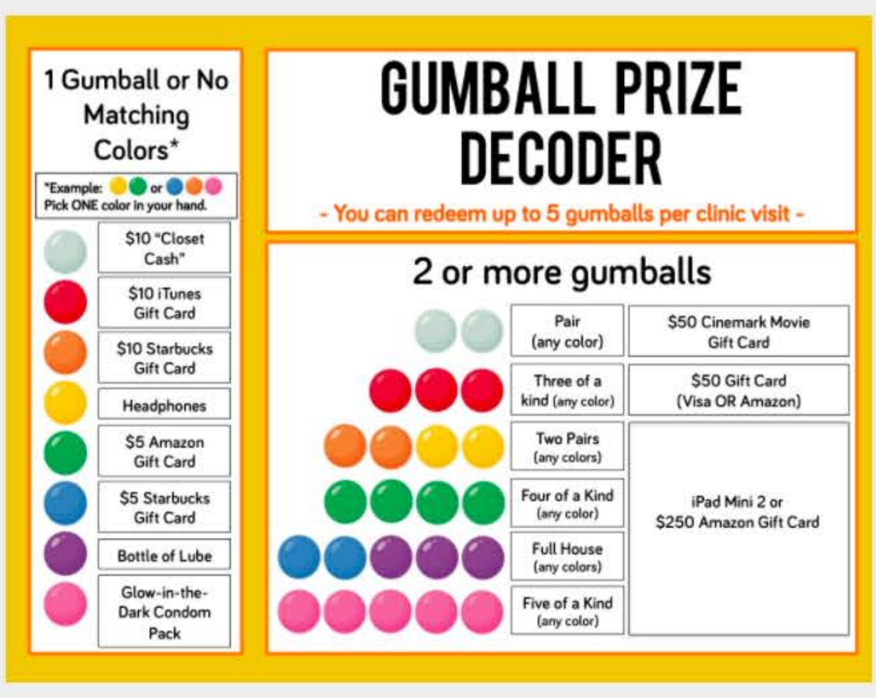

We know you'll want all the points you can get so, we uploaded the newest quiz: **Knot Your Daddy's Quiz!** Brush up on that **BDSM** trivia knowledge and get up to 200 more points!

HOW IT WORKS

You need 500 points to earn a gumball. The more gumballs you earn, the higher your chance for larger prizes! There are several ways to earn points with Stick To It:

- After you've signed up, set a date for your next STD/HIV test. This will start a timer to remind you to test every 3 months.
- **Earn points!** Between now and your next test, you can earn points for responding to online quizzes. We'll notify you whenever we've uploaded a new quiz. You can also earn points for every friend you invite who signs up for Stick To It.
- **Get tested!** Pop into the AHF Wellness Centers located in Oakland or Hollywood and get tested. You can redeem your points for gumballs at the clinic which gives you a chance to win prizes!
- You can win prizes worth up to $250.

HOW TO EARN POINTS

- Register: 200 points
- Set your testing timer: 150 points
- Check out How It Works: 100 points
- Answer a quiz correctly: up to 200 points
- Get a friend to join Stick To It: 150 points
- Get tested for STDs and/or HIV: 500 points every 3 months
- You'll get points for any test result! If your STD tests are negative, you will earn an additional 500 points. If you test positive, no problem! Drop back in to the clinic for STD treatment, and you will still get 500 additional points! Regardless of your test results, you will be rewarded for taking care of your health!

INVITE FRIENDS!

Invite a friend to join Stick To It. When they join you'll get 150 points. More friends, more points! Plus, you're helping your friends take care of their health - even sweeter!

SEND THEM AN EMAIL

SEND

WHERE TO TEST

To redeem your points for a chance at prizes, get tested for STDs/ HIV at one of these two locations:

Oakland

- **Clinic Name:** AHF Wellness Center
- **Address:** 238 E 18th St., Oakland, CA 94606 (Inside the Out of the Closet Thrift Store)
- **Days Open/Hours:** Drop-In Hours: Monday & Thursday 2:00-6:30pm
- **Phone:** (510) 251-8678
- **Transportation routes:** Accessible transportation includes the Lake Merritt Bart Station (about a mile from the clinic) Or several AC Transit Bus Routes including: 18, 14, 1/1R, 62, and 26 within close proximity. Find it on 511.org
- **Parking:** Out of the Closet parking lot (free), metered parking on street

Los Angeles

- **Clinic Name:** AHF Hollywood Wellness Center
- **Address:** 1300 N Vermont Ave, Doctor's Tower Suite 407, Los Angeles, CA 90027
- **Days Open/Hours:** Monday Wednesday Thursday Friday 5:30pm - 8pm (space is limited, please arrive between 4pm and 5pm). Saturday 9:30am - 4pm (space is limited, please arrive before 1pm).
- **Phone:** (866) 339-2525
- **Transportation routes:** Metro Red Line to Vermont/Sunset Find it on go511.com
- **Parking:** Enter parking lot from Fountain Ave or Vermont Ave onto De Longpre Ave. (parking is FREE with validation).

CONTACT US

If you want to know more about our team, check out our page here!

Principal Investigator:
Sandra I. McCoy, PhD, MPH
(510) 642-0513
smccoy@berkeley.edu

Project Coordinator (Northern California):
Reva Grimball
(650) 466-8711
rgrimball@berkeley.edu

Project Coordinator (Southern California):
Lauren Natoli
(323) 515-9586
lauren.natoli@aidshealth.org

UC Berkeley's Committee for Protection of Human Subjects
(510) 642-7461
subjects@berkeley.edu
