# Supplementary material for: An Intervention Using Gamification to Increase Human Immunodeficiency Virus and Sexually Transmitted Infection Screening Among Young Men Who Have Sex With Men in California: Rationale and Design of Stick To It
Source: JMIR Res Protoc. 2017 Jul 17;6(7):e140. doi: 10.2196/resprot.8064 (PMC5537559; doi:10.2196/resprot.8064)
Supplement: Multimedia Appendix 3 [file resprot_v6i7e140_app3.pdf]

ARE YOU 18-26?

Earn points! Get tested for free! Win prizes!

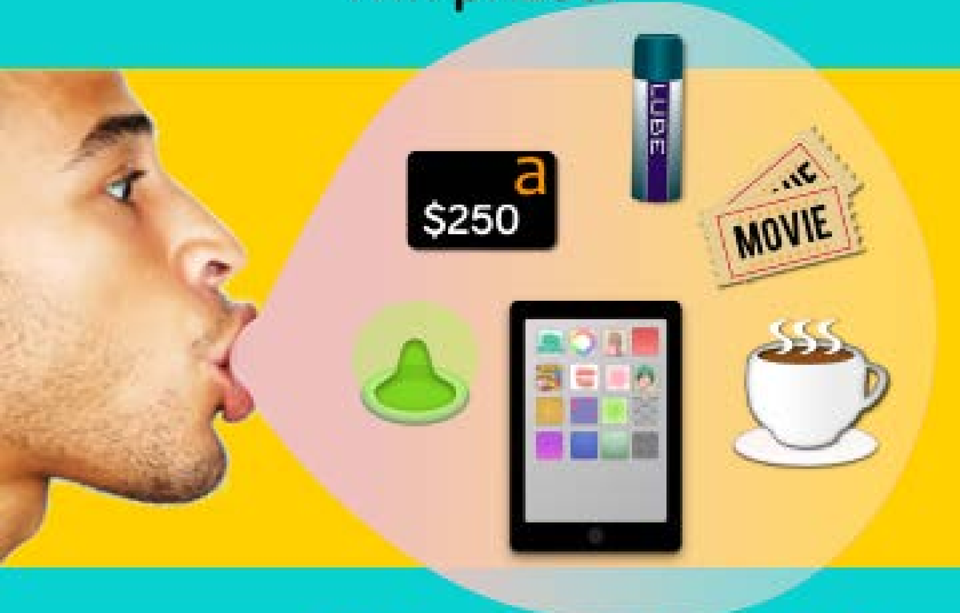

JOIN *Stick To It!*

Check out this research project

@ stick2it.org

AHF

Berkeley

David Geffen School of Medicine

A few winning mouthfulls...

- 2 OF A KIND
- A FULL MOUTH
- 3 OF A KIND
- CHEW PAIRS
- 4 OF A KIND
- A POP!
- A RAINBLOW
- THE MAGIC GUMBALL
- THE GUM-HIT WONDER

Get points, get gumballs, get prizes!

Stick To It!

Get tested for STDs/HIV and win prizes. We'll help you stick to a regular schedule!

Check out this research project @

stick2it.org

Berkeley School of Public Health

UCLA David Geffen School of Medicine

AHF AIDS HEALTHCARE FOUNDATION

18-26 gay / bi / queer guy?

Join

Stick To It!

Get tested for STDs/HIV and win prizes!

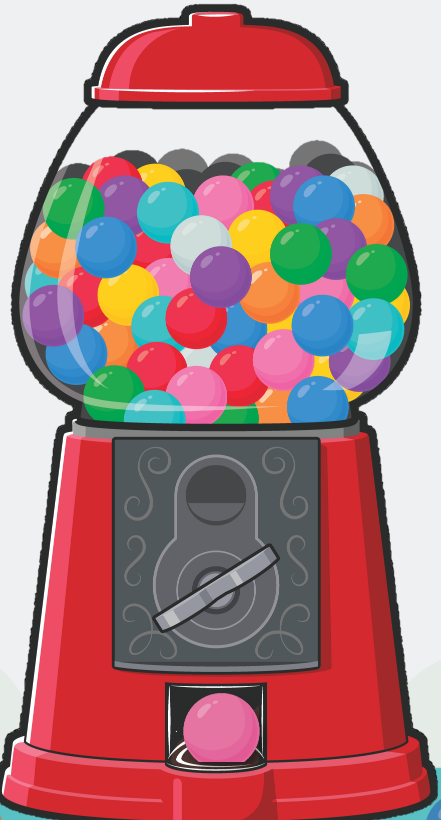

We'll help you stick to a regular schedule!

Check out this research project

@ stick2it.org

Berkeley School of Public Health

UCLA David Geffen School of Medicine

AHF AIDS HEALTHCARE FOUNDATION

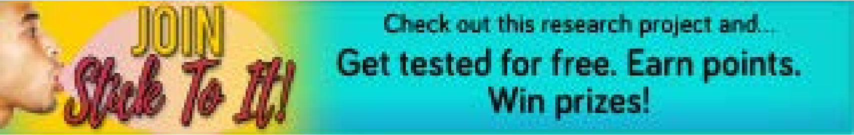
JOIN
Stick To It!
Check out this research project and...
Get tested for free. Earn points.
Win prizes!
